# Development of Prognostic Indicator Based on Autophagy-Related lncRNA Analysis in Colon Adenocarcinoma

**DOI:** 10.1155/2020/9807918

**Published:** 2020-09-02

**Authors:** Weige Zhou, Shijing Zhang, Hui-biao Li, Zheyou Cai, Shuting Tang, Li-xia Chen, Jian-ying Lang, Zheng Chen, Xin-lin Chen

**Affiliations:** ^1^School of Basic Medical Science, Guangzhou University of Chinese Medicine, Guangzhou, China; ^2^The First Affiliated Hospital, Guangzhou University of Chinese Medicine, Guangzhou, China; ^3^The First Clinical College, Guangzhou University of Chinese Medicine, Guangzhou, China; ^4^Department of Stomatology, The Third Affiliated Hospital of Sun Yat-sen University, Guangzhou, China

## Abstract

There were no systematic researches about autophagy-related long noncoding RNA (lncRNA) signatures to predict the survival of patients with colon adenocarcinoma. It was necessary to set up corresponding autophagy-related lncRNA signatures. The expression profiles of lncRNAs which contained 480 colon adenocarcinoma samples were obtained from The Cancer Genome Atlas (TCGA) database. The coexpression network of lncRNAs and autophagy-related genes was utilized to select autophagy-related lncRNAs. The lncRNAs were further screened using univariate Cox regression. In addition, Lasso regression and multivariate Cox regression were used to develop an autophagy-related lncRNA signature. A risk score based on the signature was established, and Cox regression was used to test whether it was an independent prognostic factor. The functional enrichment of autophagy-related lncRNAs was visualized using Gene Ontology and Kyoto Encyclopedia of Genes and Genomes. Ten prognostic autophagy-related lncRNAs (AC027307.2, AC068580.3, AL138756.1, CD27-AS1, EIF3J-DT, LINC01011, LINC01063, LINC02381, AC073896.3, and SNHG16) were identified to be significantly different, which made up an autophagy-related lncRNA signature. The signature divided patients with colon adenocarcinoma into the low-risk group and the high-risk group. A risk score based on the signature was a significantly independent factor for the patients with colon adenocarcinoma (HR = 1.088, 95%CI = 1.057 − 1.120; *P* < 0.001). Additionally, the ten lncRNAs were significantly enriched in autophagy process, metabolism, and tumor classical pathways. In conclusion, the ten autophagy-related lncRNAs and their signature might be molecular biomarkers and therapeutic targets for the patients with colon adenocarcinoma.

## 1. Introduction

Colorectal cancer (CRC) ranked third in incidence and second in mortality of all types of cancers worldwide [1]. It is estimated that more than 1.4 million new cases will be diagnosed with CRC, and approximately 53,200 will die of the disease in 2020 [2]. Antineoplastic protocols included endoscopic and surgical local excision, radiotherapy and systemic treatment, local ablative therapies, targeted therapy, immunotherapy, and palliative chemotherapy [3]. Although these treatments had dramatic progress, the 5-year relative survival rate for colon cancer was 64% [2]. Colon adenocarcinoma (COAD) is the most ordinary histological subtype of CRC [4].

Autophagy, a multistep lysosomal degradation process which promoted metabolic adaptation and nutrient circulation, has been widely studied and demonstrated to be involved in cancer development [5]. In both physiological and pathological situations, autophagy is central to the maintenance of organismal homeostasis. Accordingly, disruption of autophagy is highly related to clinically relevant diseases as diverse as cancer, neurodegenerative disease, pathogen infection, and heart disease [6]. Recently, a large number of findings revealed that autophagy had multiple functions in occurrence, maintenance, and development of tumors [7]. Genetic evidence suggested that autophagy was a tumor-suppressor mechanism, and it was also clear that autophagy could promote tumor survival in the response to chemotherapy and under stressful conditions [8]. For the past few years, some researchers strived to find new targeted therapeutic strategies for colon adenocarcinoma by investigating autophagy pathways [9].

Long noncoding RNAs (lncRNAs) were found to perform a wide range of functions in various important biological processes, including cell proliferation and differentiation, genetic regulation of gene expression, RNA attenuation, RNA splicing, protein folding, and microRNA (miRNA) regulation [10]. In CRC, autophagy was known to have dual and contradictory roles in carcinogenesis, but the precise mechanisms resulting in autophagy in cancer were not yet fully verified [11, 12]. lncRNAs were involved in the development, invasion and metastasis, prognosis, and the chemoresistance of colon adenocarcinoma via modulating autophagy [13–16]. These studies focused on single or a few lncRNAs for colon adenocarcinoma [13–16]. The lncRNAs' expression profiles of TCGA database were not performed to explore novel biomarkers for forecasting the prognosis of colon adenocarcinoma. Therefore, we aimed to utilize TCGA database to establish autophagy-related lncRNA signatures and seek new biomarkers to predict the prognosis of the patients with colon adenocarcinoma.

## 2. Materials and Methods

### 2.1. Datasets and Sample Extraction

The RNA sequencing (RNA-seq) data about colon adenocarcinoma from The Cancer Genome Atlas (TCGA) database (https://portal.gdc.cancer.gov/) were obtained. The inclusion criteria were set as follows: (1) patients were diagnosed as colon adenocarcinoma; (2) patients had complete lncRNA data and clinical information. According to the inclusion criteria, 480 patients with colon adenocarcinoma were included. In addition, the patient's complete clinical information was downloaded from TCGA. While screening clinical information, samples with a follow-up time of less than 30 days were excluded. Since the data involved in this study were all from the TCGA database and strictly follow the TCGA publication guidelines (http://cancergenome.nih.gov/abouttcga/policies/publicationguidelines), there is no need for approval by the ethics committee.

### 2.2. Screening of lncRNAs and Autophagy-Related Genes

The profiles of lncRNAs were acquired from all the RNA-seq dataset. The total RNA expression data were standardized through log2 transformation. The autophagy-related gene list was downloaded from the Human Autophagy Database (HADb, http://autophagy.lu/clustering/index.html). The correlation between lncRNAs and autophagy-related genes was calculated using the Pearson correlation. The square of correlation coefficient ∣*R*^2^ | >0.3 and *P* < 0.001 was considered to be autophagy-related lncRNAs. Finally, Cytoscape software 3.7.2 was used to visualize coexpression networks.

### 2.3. Identification of Prognostic Autophagy-Related lncRNAs

In the first place, the prognostic value of autophagy-related lncRNAs was assessed by univariate Cox regression. Autophagy-related lncRNAs with *P* < 0.05 in univariate analysis were incorporated into least absolute shrinkage and selection operator (Lasso) regression. Then, the results of Lasso were included into a multivariate Cox model in order to establish a risk score. We constructed a risk score based on a linear combination of the autophagy-related lncRNA expression levels multiplied with a regression coefficient (*β*): risk score = ∑_*i*=1_^*n*^*β*_*i*_∗(expression of lncRNA_*i*_). Based on the median risk score, the patients were classified into two groups: high-risk and low-risk groups. The survival differences between the two groups were compared using the log-rank test.

### 2.4. Development of Prognostic Model

Cox regression was utilized to build an independent prognostic model. Nomogram was utilized to predict survival for the patients. The index of concordance (*C*-index), calibration curves, and receiver operating characteristic (ROC) curves were applied to explore the accuracy of the model. The demographic data were included in multivariate Cox regression in order to confirm whether the risk score was an independent indicator for the prognosis.

### 2.5. Functional Analysis

Gene set enrichment analysis (GSEA, http://www.broadinstitute.org/gsea/index.jsp) was utilized to interpret the functional enrichment of gene expression data. We explored the functional enrichment of autophagy-related lncRNAs with a prognostic value and visualized the top 5 Gene Ontology (GO) and the Kyoto Encyclopedia of Genes and Genomes (KEGG) pathways related to autophagy.

### 2.6. Statistical Analysis

The Kaplan-Meier method was utilized to generate the survival curves, and the log-rank test was used for comparison. Cox regression and Lasso regression were utilized to estimate the prognostic impact of the autophagy-related lncRNA signature and clinicopathological data. The statistical analyses were conducted in R language (version 3.6). Statistical tests were bilateral, with *P* ≤ 0.05 being statistically significant.

## 3. Results

### 3.1. Construction of a Coexpression Network

A total of 14,142 lncRNAs were identified in TCGA-COAD. A total of 257 autophagy-related genes were obtained from HADb, among which 210 genes were expressed in colon adenocarcinoma (Table S1). An autophagy-related gene lncRNA coexpression network was constructed to identify the autophagy-related lncRNAs. Finally, 1,281 autophagy-related lncRNAs were selected (∣*R*^2^ | >0.3 and *P* < 0.001).

### 3.2. Identification of Prognostic Autophagy-Related lncRNA Signature

**Figure 1:**
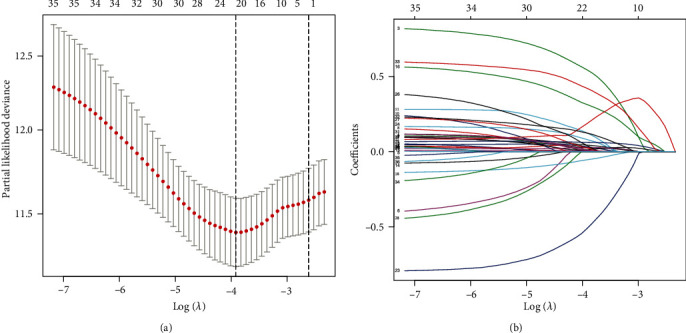
Autophagy-related lncRNA selection utilizing Lasso model. (a) Lasso coefficient values of 21 autophagy-related lncRNAs in colon adenocarcinoma. The vertical dashed lines are at the optimal log (lambda) value. (b) Profiles of Lasso coefficients.

**Figure 2:**
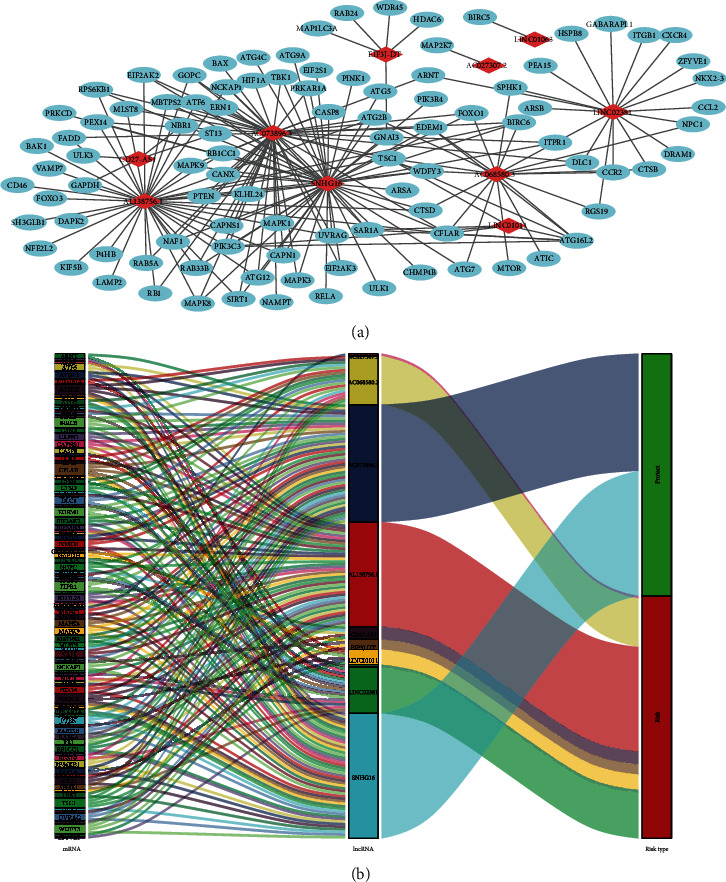
The coexpression network and Sankey diagram of prognostic autophagy-related lncRNAs. (a) The coexpression network between prognostic lncRNAs and autophagy-related genes in colon adenocarcinoma. Red diamond nodes represent prognostic lncRNAs, and the sky blue round nodes represent autophagy-related genes. The coexpression network was visualized using Cytoscape 3.7.2 software. (b) Sankey diagram showed the association between prognostic autophagy-related lncRNAs, autophagy-related genes, and risk types.

**Figure 3:**
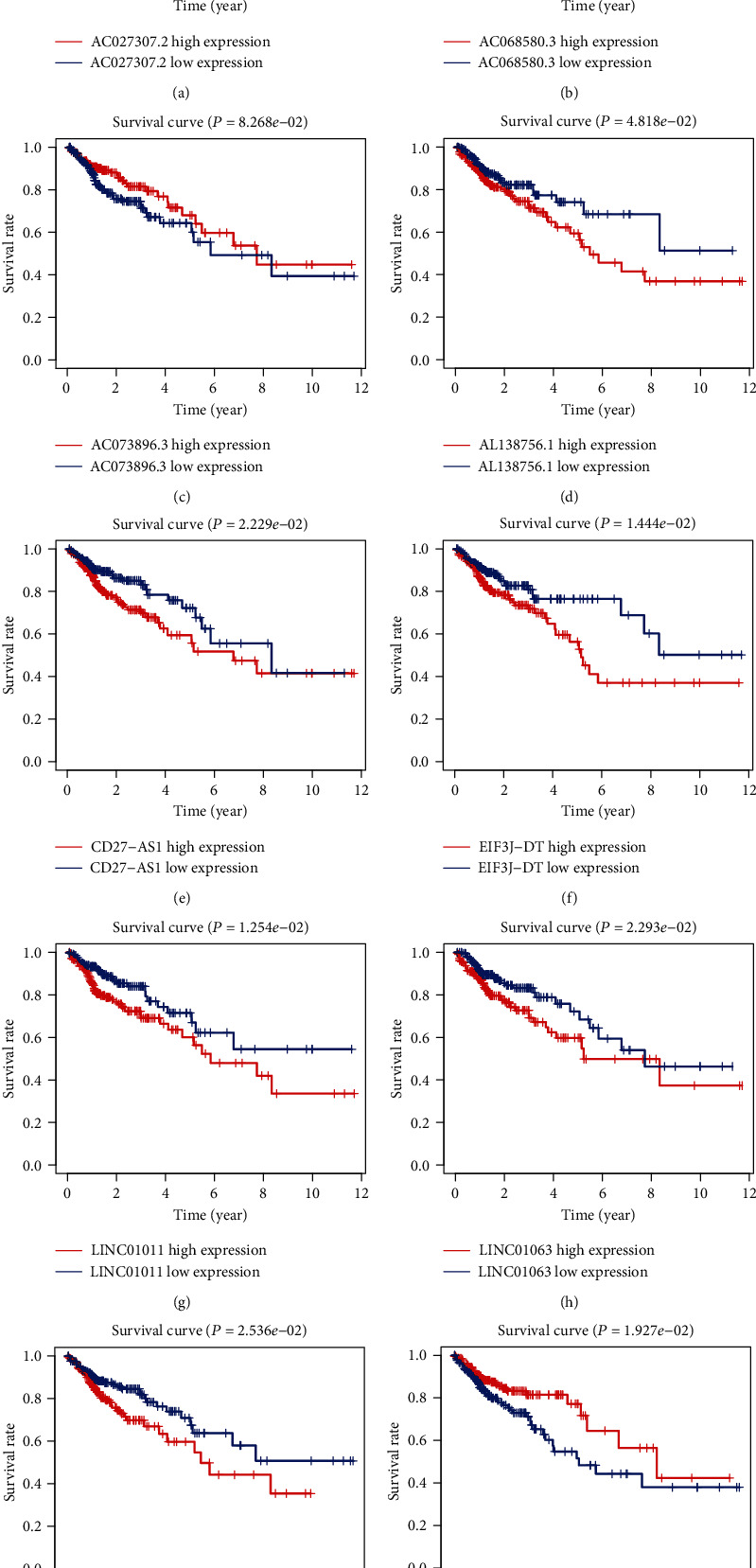
The KM survival curves of ten prognostic autophagy-related lncRNAs. Eight autophagy-related lncRNAs (AC027307.2, AC068580.3, AL138756.1, CD27-AS1, EIF3J-DT, LINC01011, LINC01063, and LINC02381) were independent unfavorable factors. Two lncRNAs (AC073896.3 and SNHG16) were independent beneficial factors for colon adenocarcinoma.

According to the results of univariate Cox, 48 autophagy-related lncRNAs had a prognostic value for the patients with colon adenocarcinoma (*P* < 0.05, Table S2). Subsequently, 21 autophagy-related lncRNAs were identified after Lasso regression (Figure 1 and Table S3). Multivariate Cox regression found that ten lncRNAs were independent prognostic factors (Figure 2). Among them, eight lncRNAs (AC027307.2, AC068580.3, AL138756.1, CD27-AS1, EIF3J-DT, LINC01011, LINC01063, and LINC02381) were harmful prognostic factors, and the others (AC073896.3 and SNHG16) were favorable prognostic factors (Table 1 and Figure 3). These ten lncRNAs were utilized to establish an autophagy-related lncRNA signature. The formula of the risk score was as follows: risk score = (0.09742∗AC027307.2) + (0.89061∗AC068580.3) + (0.27224∗LINC01011) + (0.53477∗EIF3J‐DT) − (0.13154∗SNHG16) − (0.93184∗AC073896.3) + (0.20468∗AL138756.1) + (0.08307∗CD27‐AS1) + (0.20329∗LINC02381) + (0.57267∗LINC01063).

### 3.3. The Prognostic Influence of the Established Signature

**Figure 4:**
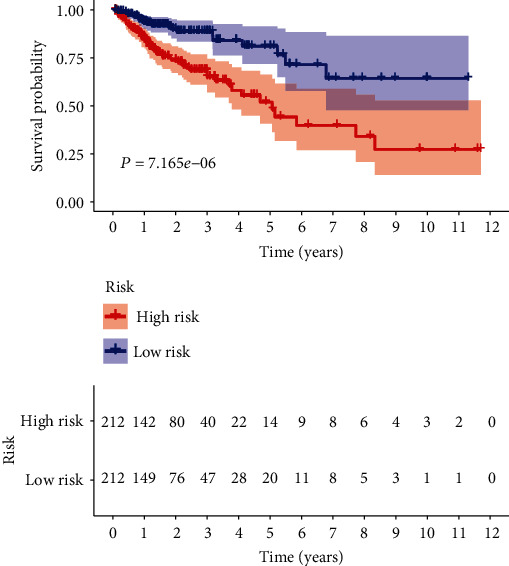
The KM survival curve of risk score based on ten autophagy-related lncRNAs.

**Figure 5:**
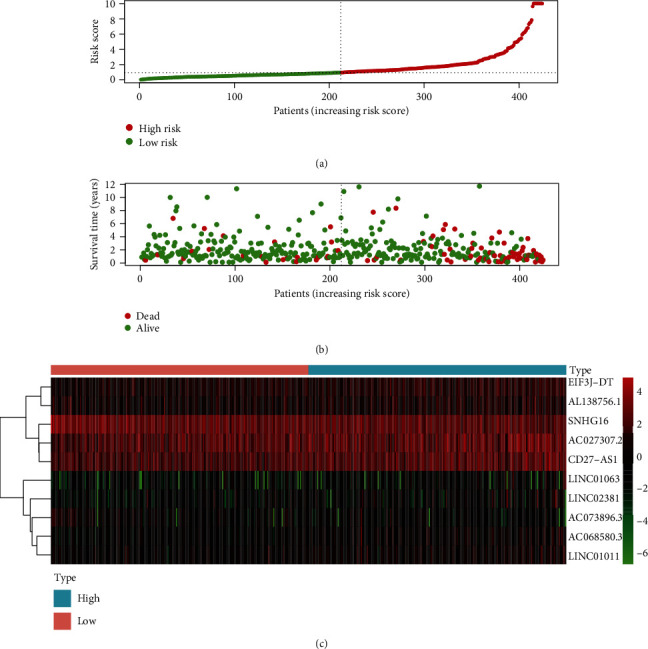
The analysis of autophagy-related lncRNA signature for patients with colon adenocarcinoma. (a) The risk score between the high-risk group and the low-risk group. (b) The survival time of the patients. (c) Heat map of ten autophagy-related lncRNAs' expression. The color from green to red reveals a rising tendency from low to high levels.

The risk score was significantly associated with the overall survival (OS) of patients with colon adenocarcinoma. The high-risk group had shorter OS compared with the low-risk group (*P* = 7.165*e* − 06, log-rank test) (Figure 4). Cox regression indicated significant prognostic impact of the risk score for the patients with colon adenocarcinoma (Figure 5).

### 3.4. Clinical Value of the Autophagy-Related lncRNA Signature

**Figure 6:**
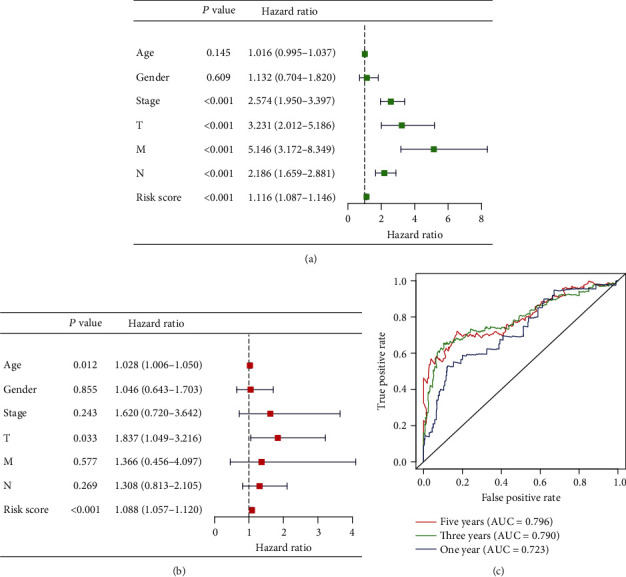
Prognostic indicators based on autophagy-related lncRNAs showed great predictive performance. The forest plots for univariate (a) and multivariate (b) Cox regression analysis in colon adenocarcinoma. (c) The areas under the ROC curve about 1 year, 3 years, and 5 years.

**Figure 7:**
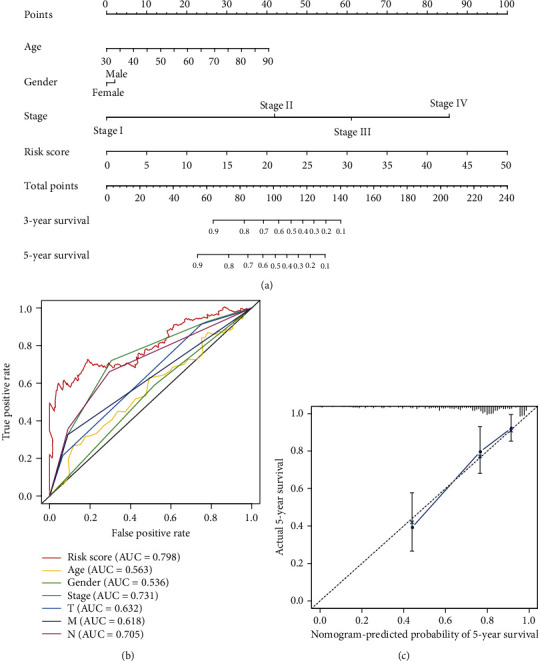
The evaluation of prognostic models based on ten autophagy-related lncRNAs. (a) The nomogram of 3-year or 5-year OS based on risk score, age, and TNM stage. (b) The ROC curves analysis based on risk score and the clinicopathologic parameters; (c) calibration plots for evaluating the agreement between the predicted and the actual OS for the prognosis model. The 45° reference line indicates perfect calibration, where the predicted probabilities are consistent with the actual probabilities.

Univariate Cox regression revealed that risk score and stage were independent prognostic indicators, and HR of risk score was 1.116 (95% CI: 1.087–1.146, *P* < 0.001, Table S4, Figure 6(a)). After controlling clinical features, risk score remained an independent prognostic indicator in multivariate analysis (HR = 1.088, 95%CI = 1.057 − 1.120, *P* < 0.001, Table 2, Figure 6(b)). The areas under the ROC curve corresponding to 1 year, 3 years, and 5 years of survival were 0.723, 0.790, and 0.796, respectively (Figure 6(c)). Risk score, age, and TNM stage were included in the nomogram. As indicated in the nomogram, risk score and TNM stage were the largest contribution to 3- and 5-year OS of patients with colon adenocarcinoma (Figure 7(a)). The *C*-index of the prognostic model was 0.796 (95% CI: 0.739-0.853). The AUC of five-year survival rate showed that risk score (0.798) and stage (0.731) had a certain prediction ability (Figure 7(b)). The risk scores increased with stage, demonstrating that this autophagy-related lncRNA signature may be related to the progression of colon adenocarcinoma (Table 3).

### 3.5. Functional Analysis

**Figure 8:**
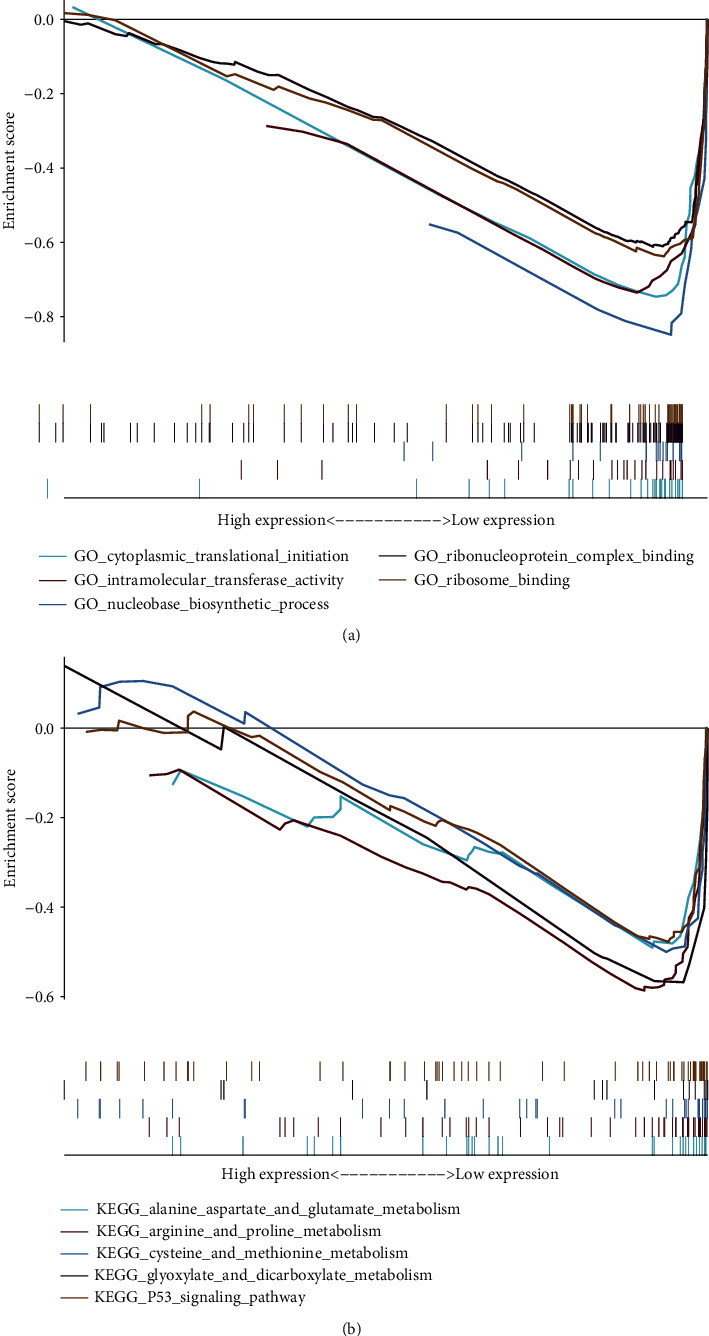
The results of functional analysis based on autophagy-related lncRNAs. (a) GO enrichment analysis; (b) KEGG enrichment analysis.

A total of 263 GO (Table S5) terms and 91 KEGG pathways were obtained (Table S6). In GO analysis, the autophagy-related lncRNAs were mainly concentrated in biological processes such as transporting damaged, denatured, or senescent proteins in the cell and organelles to the lysosome for digestion and degradation (Figure 8(a)). KEGG pathways revealed that the lncRNAs were mostly concentrated in the pathway of tumor classical pathways and metabolism (Figure 8(b)). Furthermore, we also found that the gene sets were connected with vital biological processes and functional pathways of tumorigenesis and cancer progression. For instance, leukocyte transendothelial migration, angiogenesis, and hypoxia were closely associated with the invasion and metastasis of cancer.

## 4. Discussion

Autophagy is a highly regulated process that degrades and recycles cellular components [17]. Dysregulation of autophagy was implicated in many diseases [18]. As a large and heterogeneous subclass of ncRNAs, lncRNAs played an indispensable role in different aspects of tumorigenesis which were considered a new type of biomarkers in cancer diagnosis and prognosis [19]. Most researches focused on the function of specific genes involved in autophagy [20–22]. There are no systematic studies about autophagy-related lncRNA signatures to predict the survival of patients with colon adenocarcinoma. Thus, it was necessary to establish an autophagy-related lncRNA signature to predict the prognosis of patients with colon adenocarcinoma based on the large-scale databases.

In this study, autophagy-related lncRNAs were screened by constructing a coexpression network of lncRNA and autophagy-related genes. Further, Lasso regression and Cox regression were utilized to obtain the following 10 prognostic autophagy-related lncRNAs: AC027307.2, AC068580.3, AL138756.1, CD27-AS1, EIF3J-DT, LINC01011, LINC01063, LINC02381, AC073896.3, and SNHG16. The ten autophagy-related lncRNAs might be prognostic molecular markers of prognosis and potential therapeutic targets for the patients with colon adenocarcinoma.

Six autophagy-related lncRNAs (SNHG16, EIF3J-DT, CD27-AS1, LINC01063, LINC01011, and LINC02381) were reported to be associated with cancer. (1) SNHG16 promoted Hep3B/So cell viability, autophagy, and suppress apoptosis to maintain its resistance to sorafenib by regulating miR-23b-3p [23]. SNHG16 promoted proliferation, migration, invasion, and autophagy of neuroblastoma cells through sponging miR-542-3p and upregulating autophagy-related gene 5 (ATG5) [24]. SNHG16 promoted progression of osteosarcoma and improved cisplatin resistance by sponging miR-16 to upregulate autophagy-related 4B [25]. (2) EIF3J-DT which was also named EIF3J-AS1 has been widely studied and found to be related to many kinds of cancers. Liu et al. revealed that EIF3J-DT could promote proliferation and reduce apoptosis in CRC cells, revealing that EIF3J-DT was a risk factor and possessed oncogenic functions in CRC [26]. EIF3J-DT inversely regulated miR-122e-5p via acting as a competing endogenous RNA in hepatocellular carcinoma (HCC) cells [27]. EIF3J-DT was significantly upregulated in HCC and closely correlated with poor prognosis [28]. Additionally, EIF3J-DT could be used as a therapeutic target and a potential biomarker for the diagnosis and prognosis of buccal mucosa squamous cell carcinoma [29]. (3) Ma et al. found that CD27-AS1 served as a carcinogenic RNA and the regulatory role of CD27-AS1 on CD27 contributes to the melanomagenesis [30]. (4) It was found that increased expression level of LINC01063 was significantly correlated with metastasis and poor prognosis of the patients with colon adenocarcinoma [31]. (5) Fan et al. found that LINC01011 controlled cisplatin sensitivity and mitochondrial fission via suppressing BRCA1 transcription in a tongue SCC model [32]. (6) LINC02381 might have inhibiting effects on CRC tumorigenesis partly through regulating PI3K pathway [33].

For the four remaining autophagy-related lncRNAs (AC027307.2, AC068580.3, AL138756.1, and AC073896.3), there were no studies to report their prognostic roles in cancer. Thus, more researches were necessary to explore how these lncRNAs affect the prognosis of patients with colon adenocarcinoma through autophagy exactly.

A signature based on 10 autophagy-related lncRNAs significantly predicted the prognosis of colon adenocarcinoma patients. Consistent with previous studies, the low-risk group had longer OS than the high-risk group [34, 35]. The areas under the ROC curve corresponding to 1 year, 3 years, and 5 years of survival were 0.723, 0.790, and 0.796. This result indicated that risk score signature had a certain potential in predicting survival. Both univariate and multivariate Cox analyses revealed that the signature could be used as an independent prognostic indicator. According to the results of *C*-index, ROC curve, and Calibration curve, the model possessed better discrimination and accuracy, revealing that it might serve as a potential predictive tool for patients with colon adenocarcinoma.

The results of functional enrichment analysis indicated that these prognostic autophagy-related lncRNAs were significantly enriched in biological processes such as autophagy-related, metabolism, and tumor-related pathways. Further, the most important pathways were enriched in autophagy processes in KEGG analysis which involved p53 classical pathways in colon adenocarcinoma. These results help us to explore the mechanism of autophagy-related lncRNAs. The previous study indicated that inhibition of autophagy in CRC cells led to antitumor effects via strengthened apoptosis through p53 and UPR activation [36]. Recently, protopin was proved to exert its antiproliferative activity through stimulating the p53 pathway [37]. There were some research reports on the molecular mechanism of amino acid metabolism and autophagy in colon adenocarcinoma. Amino acids were found to inhibit Raf-1 activation, which interfered with ERK1/2-dependent autophagy control in colon cancer HT-29 cells [38]. Targeted suppression of glutamine metabolism could inhibit the occurrence of colorectal cancer especially when unified with extracellular asparagine depletion and autophagy inhibition [39].

Several limitations existed in the current study. First, the data source of this study is single, and the amount of data included is not large, so the analysis results may have certain deviation. Second, our study is a retrospective study, and more prospective studies will be required to prove the prognostic function of autophagy-related signals. Third, in order to ensure the robustness of the prognostic model, the prognostic model of our established model is required to be further confirmed in other independent cohorts to ensure its robustness. Fourth, the functional experiments should be conducted to further indicate the potential molecular mechanisms for predicting the effect of autophagy-related lncRNAs.

In conclusion, an autophagy-related lncRNA coexpression network provided a valuable source for revealing autophagy-related lncRNA functions in colon adenocarcinoma. Ten autophagy-related lncRNAs were considered to be significantly associated with survival of the patients with colon adenocarcinoma. An autophagy-related lncRNA signature which was composed of ten autophagy-related lncRNAs was used to differentiate patients at different risks, and it was a significantly independent factor for the patients with colon adenocarcinoma. Therefore, the ten autophagy-related lncRNAs and their signature might be molecular biomarkers and therapeutic targets for the patients with colon adenocarcinoma.

## Figures and Tables

**Table 1:** Multivariate Cox results of lncRNAs based on TCGA-COAD data.

lncRNA	Coefficient	HR	95% CI of HR
AC027307.2	0.097	1.102	0.986-1.198
AC068580.3	0.891	2.437	1.378-3.469
AC073896.3	-0.932	0.394	0.231-0.707
AL138756.1	0.205	1.227	0.960-1.455
CD27-AS1	0.083	1.087	0.979-1.179
EIF3J-DT	0.535	1.707	1.153-2.256
LINC01011	0.272	1.313	0.917-1.987
LINC01063	0.573	1.773	1.261-2.560
LINC02381	0.203	1.225	0.947-1.575
SNHG16	-0.132	0.877	0.767-1.035

**Table 2:** Clinical characteristics and risk scores of colon adenocarcinoma using multivariate Cox regression.

Variable	*B*	SE	*Z*	HR	HR.95L	HR.95H	*P* value
Age	0.027	0.011	2.516	1.028	1.006	1.050	0.012
Gender	0.045	0.248	0.182	1.046	0.643	1.703	0.855
Stage	0.482	0.413	1.167	1.620	0.720	3.642	0.243
T	0.608	0.286	2.127	1.837	1.049	3.216	0.033
M	0.312	0.560	0.557	1.366	0.456	4.097	0.577
N	0.268	0.243	1.105	1.308	0.813	2.105	0.269
Risk score	0.084	0.015	5.646	1.088	1.057	1.120	<0.001

**Table 3:** Clinical influences of risk score signature for TCGA-COAD data.

Clinical	*n*	Risk score		*t*	*P*
		Mean	SD		
Age					
≤65	153	1.568	2.283	-0.566	0.572
>65	225	1.742	3.708		
Gender					
Female	175	1.595	3.670	-0.424	0.672
Male	203	1.738	2.751		
Stage					
I-II	216	1.205	1.243	-2.921	0.004
III-IV	162	2.293	4.617		
T					
T1-2	74	1.038	0.739	-3.574	<0.001
T3-4	304	1.826	3.540		
M					
M0	317	1.383	1.793	-2.068	0.043
M1	61	3.170	6.702		
N					
N0	225	1.207	1.234	-2.928	0.004
N1-2	153	2.355	4.739		

## Data Availability

All analyzed or generated data were incorporated in this published paper and its supplementary information file.

## Supplementary Materials

Supplementary 1 Table S1: The expression profile of autophagy-related gene in colon adenocarcinoma.

Supplementary 2 Table S2 Univariate cox results of ARlncRNAs based on TCGA-COAD data.

Supplementary 3 Table S3 LASSO regression coefficients of twenty-one ARlncRNAs.

Supplementary 4 Table S4 Clinical characteristics and risk scores of colon adenocarcinoma using univariate cox regression.

Supplementary 5 Table S5 Gene set enrichment GO analysis results according to the signature of ten ARlncRNAs (Top 20 p-value).

Supplementary 6 Table S6 Gene set enrichment KEGG analysis results based on the signature of ten ARlncRNAs (Top 20 p-value).
