# Supplementary material for: Development of Prognostic Indicator Based on Autophagy-Related lncRNA Analysis in Colon Adenocarcinoma
Source: Biomed Res Int. 2020 Sep 2;2020:9807918. doi: 10.1155/2020/9807918 (PMC7486634; doi:10.1155/2020/9807918)
Supplement: Supplementary 2 — Table S2 Univariate cox results of ARlncRNAs based on TCGA-COAD data. [file 9807918.f2.docx]

Table S2 Univariate cox results of ARlncRNAs based on TCGA-COAD data.

| lncRNA | KM | B | SE | HR | HR.95L | HR.95H | p-value |
| --- | --- | --- | --- | --- | --- | --- | --- |
| AC027307.2 | 0.004 | 0.112 | 0.038 | 1.118 | 1.037 | 1.205 | 0.003 |
| ARRDC1-AS1 | 0.012 | 0.182 | 0.061 | 1.199 | 1.065 | 1.351 | 0.003 |
| AC068580.3 | 0.009 | 0.465 | 0.154 | 1.592 | 1.177 | 2.152 | 0.003 |
| AC063948.1 | 0.033 | 0.340 | 0.102 | 1.406 | 1.150 | 1.718 | 0.001 |
| PCAT6 | 0.039 | 0.167 | 0.056 | 1.181 | 1.059 | 1.317 | 0.003 |
| AC040977.1 | 0.001 | 0.174 | 0.075 | 1.190 | 1.028 | 1.377 | 0.020 |
| AC022150.2 | 0.038 | 0.175 | 0.084 | 1.191 | 1.010 | 1.404 | 0.038 |
| AC068888.1 | 0.043 | 0.401 | 0.187 | 1.493 | 1.034 | 2.155 | 0.032 |
| AC008280.3 | 0.010 | 0.426 | 0.192 | 1.531 | 1.050 | 2.232 | 0.027 |
| AC105219.1 | 0.033 | 0.135 | 0.062 | 1.145 | 1.013 | 1.293 | 0.030 |
| AL161729.4 | 0.002 | 0.335 | 0.110 | 1.398 | 1.127 | 1.734 | 0.002 |
| AL354993.2 | 0.001 | 0.219 | 0.094 | 1.245 | 1.036 | 1.495 | 0.019 |
| AC007128.1 | 0.036 | 0.386 | 0.181 | 1.471 | 1.032 | 2.097 | 0.033 |
| AC087481.3 | 0.035 | 0.199 | 0.080 | 1.220 | 1.043 | 1.427 | 0.013 |
| LINC01011 | 0.013 | 0.408 | 0.156 | 1.504 | 1.109 | 2.041 | 0.009 |
| AC020558.2 | 0.038 | 0.403 | 0.184 | 1.496 | 1.043 | 2.146 | 0.029 |
| ELFN1-AS1 | 0.030 | 0.024 | 0.010 | 1.025 | 1.005 | 1.045 | 0.015 |
| AC009403.1 | 0.048 | 0.135 | 0.067 | 1.144 | 1.004 | 1.305 | 0.044 |
| LBX2-AS1 | 0.001 | 0.103 | 0.045 | 1.109 | 1.014 | 1.212 | 0.023 |
| LINC00957 | 0.041 | 0.351 | 0.130 | 1.420 | 1.101 | 1.832 | 0.007 |
| AC023157.3 | 0.043 | 0.328 | 0.107 | 1.389 | 1.126 | 1.712 | 0.002 |
| NIFK-AS1 | 0.027 | 0.661 | 0.188 | 1.937 | 1.340 | 2.801 | 0.000 |
| EIF3J-DT | 0.014 | 0.448 | 0.114 | 1.565 | 1.252 | 1.956 | 0.000 |
| LINC01836 | 0.000 | 0.320 | 0.116 | 1.378 | 1.098 | 1.728 | 0.006 |
| AP001505.1 | 0.026 | 0.066 | 0.028 | 1.068 | 1.011 | 1.128 | 0.018 |
| SNHG16 | 0.019 | (0.166) | 0.065 | 0.847 | 0.745 | 0.963 | 0.011 |
| BACE1-AS | 0.023 | 0.169 | 0.075 | 1.184 | 1.023 | 1.372 | 0.024 |
| MIR4435-2HG | 0.003 | 0.231 | 0.114 | 1.259 | 1.006 | 1.576 | 0.044 |
| AC009779.2 | 0.013 | 0.217 | 0.055 | 1.242 | 1.116 | 1.383 | 0.000 |
| ZEB1-AS1 | 0.004 | 0.828 | 0.188 | 2.289 | 1.582 | 3.313 | 0.000 |
| AL132712.1 | 0.018 | 0.176 | 0.088 | 1.193 | 1.004 | 1.418 | 0.045 |
| AL138756.1 | 0.048 | 0.185 | 0.081 | 1.203 | 1.026 | 1.411 | 0.023 |
| NKILA | 0.021 | 0.224 | 0.065 | 1.250 | 1.101 | 1.420 | 0.001 |
| AC005229.4 | 0.045 | 0.460 | 0.146 | 1.584 | 1.190 | 2.109 | 0.002 |
| AP001469.3 | 0.029 | 0.433 | 0.161 | 1.542 | 1.124 | 2.115 | 0.007 |
| AC019069.1 | 0.037 | 0.205 | 0.089 | 1.228 | 1.031 | 1.462 | 0.021 |
| AC107375.1 | 0.004 | 0.273 | 0.114 | 1.314 | 1.051 | 1.644 | 0.017 |
| CD27-AS1 | 0.022 | 0.129 | 0.033 | 1.138 | 1.067 | 1.213 | 0.000 |
| AL118506.1 | 0.028 | 0.252 | 0.100 | 1.286 | 1.057 | 1.566 | 0.012 |
| AC007383.2 | 0.015 | 0.208 | 0.083 | 1.231 | 1.047 | 1.449 | 0.012 |
| AC073611.1 | 0.002 | 0.457 | 0.194 | 1.580 | 1.079 | 2.312 | 0.019 |
| AC003991.2 | 0.015 | (0.430) | 0.217 | 0.651 | 0.425 | 0.996 | 0.048 |
| AL451050.2 | 0.044 | 0.439 | 0.170 | 1.551 | 1.111 | 2.165 | 0.010 |
| AC074117.1 | 0.018 | 0.236 | 0.095 | 1.267 | 1.051 | 1.526 | 0.013 |
| LINC02381 | 0.025 | 0.218 | 0.084 | 1.243 | 1.055 | 1.466 | 0.010 |
| LINC01063 | 0.023 | 0.557 | 0.146 | 1.745 | 1.310 | 2.324 | 0.000 |
| AL162586.1 | 0.002 | 0.371 | 0.117 | 1.449 | 1.152 | 1.821 | 0.002 |
| AC102953.2 | 0.023 | 0.266 | 0.124 | 1.305 | 1.023 | 1.665 | 0.032 |
