# Supplementary material for: Development of Prognostic Indicator Based on Autophagy-Related lncRNA Analysis in Colon Adenocarcinoma
Source: Biomed Res Int. 2020 Sep 2;2020:9807918. doi: 10.1155/2020/9807918 (PMC7486634; doi:10.1155/2020/9807918)
Supplement: Supplementary 3 — Table S3 LASSO regression coefficients of twenty-one ARlncRNAs. [file 9807918.f3.docx]

Table S3 LASSO regression coefficients of twenty-one ARlncRNAs.

| lncRNA | Coefficient |
| --- | --- |
| AC027307.2 | 0.043527 |
| AC068580.3 | 0.545193 |
| AC105219.1 | 0.026025 |
| LINC01011 | 0.125089 |
| ELFN1-AS1 | 0.005728 |
| LINC00957 | 0.029081 |
| EIF3J-DT | 0.314208 |
| LINC01836 | 0.032674 |
| SNHG16 | -0.05421 |
| MIR4435.2HG | 0.009444 |
| AC009779.2 | 0.012322 |
| ZEB1-AS1 | 0.186233 |
| AC073896.3 | -0.51238 |
| AL138756.1 | 0.082163 |
| NKILA | 0.026421 |
| AC004264.1 | 0.042194 |
| AC019069.1 | 0.024335 |
| CD27-AS1 | 0.06238 |
| TNRC6C-AS1 | 0.010125 |
| LINC02381 | 0.124229 |
| LINC01063 | 0.422247 |
