# Supplementary material for: Development of Prognostic Indicator Based on Autophagy-Related lncRNA Analysis in Colon Adenocarcinoma
Source: Biomed Res Int. 2020 Sep 2;2020:9807918. doi: 10.1155/2020/9807918 (PMC7486634; doi:10.1155/2020/9807918)
Supplement: Supplementary 4 — Table S4 Clinical characteristics and risk scores of colon adenocarcinoma using univariate cox regression. [file 9807918.f4.docx]

Table S4 Clinical characteristics and risk scores of colon adenocarcinoma using univariate cox regression.

| Variable | B | SE | Z | HR | HR.95L | HR.95H | p value |
| --- | --- | --- | --- | --- | --- | --- | --- |
| age | 0.016 | 0.011 | 1.458 | 1.016 | 0.995 | 1.037 | 0.145 |
| gender | 0.124 | 0.242 | 0.511 | 1.132 | 0.704 | 1.820 | 0.609 |
| stage | 0.945 | 0.142 | 6.678 | 2.574 | 1.950 | 3.397 | <0.001 |
| T | 1.173 | 0.241 | 4.856 | 3.231 | 2.012 | 5.186 | <0.001 |
| M | 1.638 | 0.247 | 6.634 | 5.146 | 3.172 | 8.349 | <0.001 |
| N | 0.782 | 0.141 | 5.557 | 2.186 | 1.659 | 2.881 | <0.001 |
| Risk score | 0.110 | 0.014 | 8.083 | 1.116 | 1.087 | 1.146 | <0.001 |
