# Supplementary material for: Development of Prognostic Indicator Based on Autophagy-Related lncRNA Analysis in Colon Adenocarcinoma
Source: Biomed Res Int. 2020 Sep 2;2020:9807918. doi: 10.1155/2020/9807918 (PMC7486634; doi:10.1155/2020/9807918)
Supplement: Supplementary 5 — Table S5 Gene set enrichment GO analysis results according to the signature of ten ARlncRNAs (Top 20 p-value). [file 9807918.f5.docx]

Table S5 Gene set enrichment GO analysis results according to the signature of ten ARlncRNAs (Top 20 p-value).

| Name | SIZE | ES | NES | NOM  p-value | FDR  q-value | FWER  p-value | RANK  AT  MAX | LEADING EDGE |
| --- | --- | --- | --- | --- | --- | --- | --- | --- |
| GO_RIBOSOME_BINDING | 57 | -0.64751 | -2.29564 | 0.00E+00 | 0.036201 | 0.033 | 3675 | tags=51%, list=7%, signal=54% |
| GO_INTRAMOLECULAR_TRANSFERASE_ACTIVITY | 26 | -0.74938 | -2.27172 | 0.00E+00 | 0.028204 | 0.043 | 5939 | tags=65%, list=11%, signal=73% |
| GO_RIBONUCLEOPROTEIN_COMPLEX_BINDING | 129 | -0.61546 | -2.23717 | 0.00E+00 | 0.029052 | 0.056 | 4587 | tags=51%, list=8%, signal=56% |
| GO_NUCLEOBASE_BIOSYNTHETIC_PROCESS | 19 | -0.88093 | -2.15818 | 0.00E+00 | 0.065674 | 0.135 | 3082 | tags=74%, list=6%, signal=78% |
| GO_CYTOPLASMIC_TRANSLATIONAL_INITIATION | 31 | -0.7623 | -2.13789 | 0.00E+00 | 0.068613 | 0.155 | 4363 | tags=68%, list=8%, signal=74% |
| GO_TRICARBOXYLIC_ACID_CYCLE | 34 | -0.79337 | -2.12214 | 0.00E+00 | 0.070841 | 0.172 | 2514 | tags=59%, list=5%, signal=62% |
| GO_SMALL_RIBOSOMAL_SUBUNIT | 73 | -0.82031 | -2.1187 | 0.00E+00 | 0.063688 | 0.176 | 6104 | tags=79%, list=11%, signal=89% |
| GO_TRANSLATION_FACTOR_ACTIVITY_RNA_BINDING | 86 | -0.63008 | -2.10847 | 0.00E+00 | 0.063482 | 0.192 | 4580 | tags=52%, list=8%, signal=57% |
| GO_MATURATION_OF_LSU_RRNA | 20 | -0.89615 | -2.09414 | 0.00E+00 | 0.069531 | 0.219 | 3362 | tags=70%, list=6%, signal=75% |
| GO_NUCLEOID | 43 | -0.73983 | -2.08926 | 0.00E+00 | 0.066604 | 0.226 | 1651 | tags=56%, list=3%, signal=57% |
| GO_CYTOPLASMIC_TRANSLATION | 94 | -0.64888 | -2.06767 | 0.00E+00 | 0.079784 | 0.264 | 6395 | tags=64%, list=12%, signal=72% |
| GO_AMINO_ACID_ACTIVATION | 49 | -0.78492 | -2.06422 | 0.00E+00 | 0.076611 | 0.272 | 4469 | tags=69%, list=8%, signal=75% |
| GO_PROTEIN_FOLDING_IN_ENDOPLASMIC_RETICULUM | 16 | -0.84566 | -2.06243 | 0.00E+00 | 0.072485 | 0.279 | 5686 | tags=75%, list=10%, signal=84% |
| GO_TRANSLATION_INITIATION_FACTOR_ACTIVITY | 51 | -0.69127 | -2.04999 | 0.00E+00 | 0.069964 | 0.304 | 4580 | tags=59%, list=8%, signal=64% |
| GO_PEPTIDE_DISULFIDE_OXIDOREDUCTASE_ACTIVITY | 15 | -0.92713 | -2.02951 | 0.00E+00 | 0.080637 | 0.35 | 2501 | tags=80%, list=5%, signal=84% |
| GO_TRANSLATION_PREINITIATION_COMPLEX | 18 | -0.84195 | -2.00935 | 0.00E+00 | 0.083391 | 0.392 | 4580 | tags=67%, list=8%, signal=73% |
| GO_ESTABLISHMENT_OF_PROTEIN_  LOCALIZATION_TO_MITOCHONDRIAL_MEMBRANE | 18 | -0.83555 | -1.9972 | 0.00E+00 | 0.087374 | 0.422 | 3405 | tags=72%, list=6%, signal=77% |
| GO_POSITIVE_REGULATION_OF_TELOMERASE_  RNA_LOCALIZATION_TO_CAJAL_BODY | 15 | -0.95564 | -1.97629 | 0.00E+00 | 0.085469 | 0.476 | 2467 | tags=100%, list=4%, signal=105% |
| GO_EUKARYOTIC_48S_PREINITIATION_COMPLEX | 15 | -0.85594 | -1.97571 | 0.00E+00 | 0.083432 | 0.476 | 3493 | tags=67%, list=6%, signal=71% |
| GO_ORGANELLAR_SMALL_RIBOSOMAL_SUBUNIT | 28 | -0.85143 | -1.96959 | 0.00E+00 | 0.08181 | 0.496 | 5549 | tags=79%, list=10%, signal=87% |
