# Supplementary material for: Development of Prognostic Indicator Based on Autophagy-Related lncRNA Analysis in Colon Adenocarcinoma
Source: Biomed Res Int. 2020 Sep 2;2020:9807918. doi: 10.1155/2020/9807918 (PMC7486634; doi:10.1155/2020/9807918)
Supplement: Supplementary 6 — Table S6 Gene set enrichment KEGG analysis results based on the signature of ten ARlncRNAs (Top 20 p-value). [file 9807918.f6.zip › Table S6.docx]

| Name | SIZE | ES | NES | NOM  p-value | FDR  q-value | FWER  p-value | | RANK  AT  MAX | LEADING EDGE |
| --- | --- | --- | --- | --- | --- | --- | --- | --- | --- |
| KEGG_CITRATE_CYCLE_TCA_CYCLE | 30 | -0.85248 | -2.14053 | 0 | 0.018125 | | 0.018 | 2255 | tags=63%, list=4%, signal=66% |
| KEGG_ARGININE_AND_  PROLINE_METABOLISM | 51 | -0.59473 | -1.96799 | 0 | 0.056631 | | 0.119 | 5411 | tags=53%, list=10%, signal=59% |
| KEGG_PROTEIN_EXPORT | 23 | -0.77651 | -1.94356 | 0 | 0.054129 | | 0.136 | 3931 | tags=57%, list=7%, signal=61% |
| KEGG_ALANINE_ASPARTATE_  AND_GLUTAMATE_METABOLISM | 32 | -0.50663 | -1.70022 | 0.005917 | 0.102781 | | 0.479 | 4730 | tags=44%, list=9%, signal=48% |
| KEGG_AMINOACYL_TRNA_BIOSYNTHESIS | 41 | -0.78411 | -2.04275 | 0.005952 | 0.031576 | | 0.056 | 4469 | tags=66%, list=8%, signal=72% |
| KEGG_PROTEASOME | 45 | -0.72236 | -1.85265 | 0.008081 | 0.065513 | | 0.232 | 5319 | tags=67%, list=10%, signal=74% |
| KEGG_CYSTEINE_AND_  METHIONINE_METABOLISM | 34 | -0.51573 | -1.7292 | 0.008511 | 0.089307 | | 0.426 | 3535 | tags=44%, list=6%, signal=47% |
| KEGG_ONE_CARBON_POOL_BY_FOLATE | 17 | -0.72871 | -1.88533 | 0.010204 | 0.055475 | | 0.194 | 3031 | tags=53%, list=5%, signal=56% |
| KEGG_P53_SIGNALING_PATHWAY | 65 | -0.48701 | -1.77891 | 0.010549 | 0.089247 | | 0.351 | 3361 | tags=42%, list=6%, signal=44% |
| KEGG_GLYCOLYSIS_GLUCONEOGENESIS | 61 | -0.52957 | -1.76078 | 0.013944 | 0.09446 | | 0.381 | 2321 | tags=39%, list=4%, signal=41% |
| KEGG_PARKINSONS_DISEASE | 101 | -0.66731 | -1.92311 | 0.016632 | 0.052698 | | 0.149 | 4739 | tags=61%, list=9%, signal=67% |
| KEGG_CELL_CYCLE | 124 | -0.5772 | -1.89005 | 0.016807 | 0.061508 | | 0.188 | 4997 | tags=56%, list=9%, signal=62% |
| KEGG_DNA_REPLICATION | 36 | -0.74186 | -1.7964 | 0.018036 | 0.085777 | | 0.332 | 6109 | tags=83%, list=11%, signal=94% |
| KEGG_OXIDATIVE_PHOSPHORYLATION | 102 | -0.64478 | -1.82299 | 0.023013 | 0.074483 | | 0.283 | 5998 | tags=64%, list=11%, signal=71% |
| KEGG_ALZHEIMERS_DISEASE | 145 | -0.48296 | -1.73279 | 0.023109 | 0.092732 | | 0.421 | 5998 | tags=52%, list=11%, signal=58% |
| KEGG_HUNTINGTONS_DISEASE | 161 | -0.4844 | -1.74436 | 0.02459 | 0.092089 | | 0.41 | 4739 | tags=48%, list=9%, signal=53% |
| KEGG_VALINE_LEUCINE_AND_  ISOLEUCINE_DEGRADATION | 44 | -0.61734 | -1.68721 | 0.025 | 0.095443 | | 0.503 | 5536 | tags=50%, list=10%, signal=56% |
| KEGG_PYRUVATE_METABOLISM | 39 | -0.57643 | -1.75278 | 0.028807 | 0.093259 | | 0.399 | 1935 | tags=44%, list=4%, signal=45% |
| KEGG_GLYOXYLATE_AND_  DICARBOXYLATE_METABOLISM | 16 | -0.61022 | -1.63171 | 0.03012 | 0.122521 | | 0.593 | 2074 | tags=44%, list=4%, signal=45% |
| KEGG_GLUTATHIONE_METABOLISM | 49 | -0.51462 | -1.63001 | 0.03112 | 0.113808 | | 0.596 | 2719 | tags=39%, list=5%, signal=41% |

Table S6 Gene set enrichment KEGG analysis results based on the signature of ten ARlncRNAs (Top 20 p-value).
